# Extraction of CRISPR-targeted sequences from the metagenome

**DOI:** 10.1016/j.xpro.2022.101525

**Published:** 2022-07-02

**Authors:** Ryota Sugimoto, Luca Nishimura, Phuong Thanh Nguyen, Ituro Inoue

**Affiliations:** 1 Human Genetics Laboratory, National Institute of Genetics, Mishima, Shizuoka 411-8540, Japan; 2 Department of Genetics, School of Life Science, The Graduate University for Advanced Studies (SOKENDAI), Mishima, Shizuoka 411-8540, Japan

**Keywords:** Bioinformatics, Sequence analysis, Genomics, CRISPR, Systems biology

## Abstract

Homology-based search is commonly used to uncover mobile genetic elements (MGEs) from metagenomes, but it heavily relies on reference genomes in the database. Here we introduce a protocol to extract CRISPR-targeted sequences from the assembled human gut metagenomic sequences without using a reference database. We describe the assembling of metagenome contigs, the extraction of CRISPR direct repeats and spacers, the discovery of protospacers, and the extraction of protospacer-enriched regions using the graph-based approach. This protocol could extract numerous characterized/uncharacterized MGEs.

For complete details on the use and execution of this protocol, please refer to Sugimoto et al. (2021).

## Highlights

• Protocol to extract CRISPR-targeted sequences from the assembled metagenomic sequences
• Extraction of sequences from the human gut metagenome without using a reference database
• Extraction of sequences with various kinds of MGEs including unclassified ones

## Abstract (editor highlights)

Publisher’s note: Undertaking any experimental protocol requires adherence to local institutional guidelines for laboratory safety and ethics.

## Graphical abstract

**Figure:**
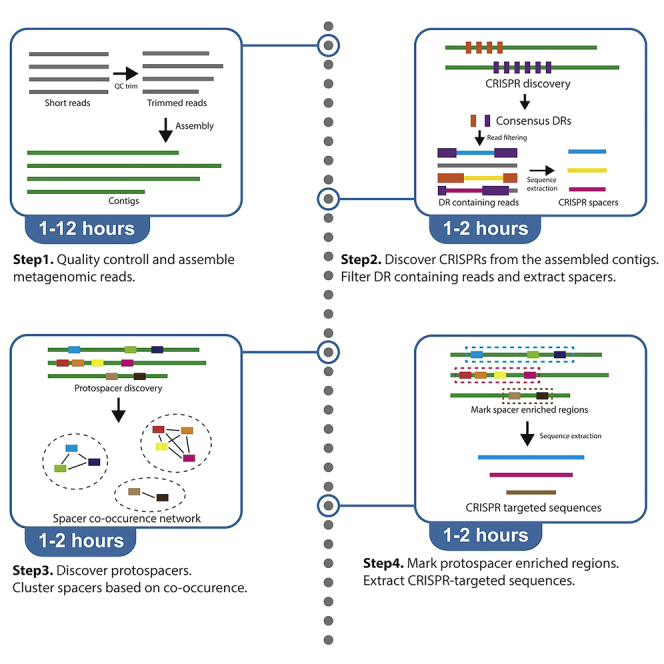

## Before you begin

### Hardware

This section describes the hardware specifications, operating system, and interpreters required to execute the protocol.

1. Computer.
  a. At least 128 GB of memory.
  b. A hyper-threading CPU with at least 8 threads.
2. Operating system.
  a. Linux distribution capable of executing scripts written in the languages of the interpreters specified below.
3. Interpreters.
  a. Python3.
  b. Java.
  c. Bash.

***Note:*** The computer must have sufficient memory for metagenome assembly. A typical human gut metagenome library ranges in size from a few GB to hundreds of GB. Therefore, assembling a large library might require more than 128 GB of memory, which is likely unavailable for a standard desktop machine. In this case, consider using high-capacity servers in a data center.

### Software

The software and python packages listed in the key resources table must be downloaded and installed on the system accordingly.

### Data collection

Metagenomic raw reads can be downloaded from numerous sources, such as Sequence Read Archive (SRA, https://www.ncbi.nlm.nih.gov/sra), Integrative Human Microbiome Projects (iHMP, https://ibdmdb.org/tunnel/public/summary.html), etc. In this article, we assume that the sequences are stored in paired short-read FASTQ files generated from whole metagenome shotgun sequencing.

***Note:*** We suggest that at least 10 independent samples from an environment are required to collect a sufficient number of spacers and candidate protospacers to build a versatile spacer co-occurrence network.

## Key resources table

**Table:** 

REAGENT or RESOURCE	SOURCE	IDENTIFIER
**Software and algorithms**		

SPAdes	(Bankevich et al., 2012)	https://github.com/ablab/spades/releases/download/v3.15.4/SPAdes-3.15.4.tar.gz
BBtools	(Bushnell, 2014)	https://sourceforge.net/projects/bbmap/
Bedtools	(Quinlan and Hall, 2010)	https://github.com/arq5x/bedtools2/releases/download/v2.30.0/bedtools-2.30.0.tar.gz
Mcl	(Enright et al., 2002)	https://micans.org/mcl/src/mcl-latest.tar.gz
Blast	(Altschul et al., 1990)	https://ftp.ncbi.nlm.nih.gov/blast/executables/blast+/LATEST/
CRISPRDetect	(Biswas et al., 2016)	https://github.com/davidchyou/CRISPRDetect_2.4
Samtools	(Danecek et al., 2021)	https://github.com/samtools/samtools/releases/download/1.15/samtools-1.15.tar.bz2
Cdhit	(Li and Godzik, 2006)	https://github.com/weizhongli/cdhit/releases/download/V4.8.1/cd-hit-v4.8.1-2019-0228.tar.gz
Biopython	(Cock et al., 2009)	https://biopython.org
NumPy	(Harris et al., 2020)	https://numpy.org
Scipy	(Virtanen et al., 2020)	https://scipy.org
Portion	N/A	https://github.com/AlexandreDecan/portion
Our custom python scripts	(Sugimoto et al., 2021)	https://doi.org/10.5281/zenodo.6621424

**Deposited data**		

Dataset from the previous study	(Sugimoto et al., 2021)	https://doi.org/10.5281/zenodo.6503687

**Other**		

Linux computer	N/A	N/A

***Note:*** Some software versions specified in this table might not be available. In such a case, use the latest version of the software and refer to their manuals.

## Materials and equipment

***Alternatives:*** Our repository also includes custom Bash scripts used in the previous study to automate the analysis pipelines. With modifications, these scripts could be used as starting points to build the custom pipeline for the reader’s purpose.

**Table:** 

Custom scripts	(Sugimoto et al., 2021)	https://doi.org/10.5281/zenodo.6621424

**CRITICAL:** These automation pipeline scripts are mainly written in Bash language (files ending their names with .sh) and require modifications to specify the file paths in their codes. The parts that require changes are noted with comments in the Bash scripts (see README files for details). Conversely, the python scripts used in this protocol do not require any change.

**Table:** 

Dataset from the previous study	(Sugimoto et al., 2021)	https://doi.org/10.5281/zenodo.6503687

***Alternatives:*** We also provide FASTA and BED-formatted files, which contain spacers, protospacers, CRISPR direct repeats, and CRISPR-targeted terminally redundant sequences extracted from approximately ten thousand human gut metagenome libraries in the previous study. This dataset includes approximately two million unique spacers, ten thousand unique CRISPR direct repeats, and approximately ten thousand CRISPR-targeted unique sequences. The protocol described in this article does not require these files. However, for the case of human gut metagenome analysis, this previously extracted dataset could be used to skip the processes and reduce the analysis time.

## Step-by-step method details

### Preprocess raw paired FASTQ files

**Timing: 1–2 h**

This process is quality control of the input sequences.

***Alternatives:*** A pipeline script for preprocessing, assembling, and spacer collection is provided in the repository (pipeline_scripts/assembly_pipeline.sh).

1. Trim poor-quality bases, adapters, and PhiX spikes.

$ bbduk.sh -Xmx100g t=8 in1=r1.fastq.gz in2=r2.fastq.gz out=trimmed.fastq.gz ftm=5 qtrim=rl trimq=20 ftl=15 ref=adapters,phix ktrim=r k=23 mink=11 hdist=1 tpe tbo maq=20

2. Remove human-derived reads.

$ bbmap.sh -Xmx100g t=8 ref={path to the human reference FASTA file} in=trimmed.fastq.gz outu=decontaminated.fastq.gz interleaved=t minratio=0.9 maxindel=3 bwr=0.16 bw=12 fast=t minhits=2 qtrim=r trimq=10 untrim=t idtag=t printunmappedcount=t kfilter=25 maxsites=1 k=14

***Note:*** We used the human genome reference FASTA file posted by the author of BBtools.

3. Correct errors in the reads.

$ tadpole.sh threads=8 -Xmx100g interleaved=t in=decontaminated.fastq.gz out=ecc.fastq.gz mode=correct

**CRITICAL:** This output FASTQ file is analysis-ready and used for assembly and spacer extraction.

### Metagenome assembly

**Timing: 1–12 h (Varies strongly depending on library)**

Assemble metagenome contigs from the preprocessed FASTQ files.

4. Assemble preprocessed FASTQ file using SPAdes.

$ spades.py -t 8 -m 100 --meta --only-assembler --12 ecc.fastq.gz -o {path to spades output directory}

***Note:*** We skipped the error-correction step in the SPAdes program to avoid the occasional endlessly running problem. Please refer to the troubleshooting section for detail.

5. Assign unique identifiers to all assembled contigs.

$ cd {path to spades output directory}

$ id=$(openssl rand -hex 12)

awk -v id=${id} '/ˆ>/ {n++; print ">contig_"id"_"n;} !/ˆ>/{print}' scaffolds.fasta > scaffolds.renamed.fasta

**CRITICAL:** Assigning unique identifiers is important to avoid conflicts in the later analyses. Here, we used randomly generated strings; however, any unique readable identifier, such as sample IDs, could be used instead.

***Optional:*** We recommend removing contigs shorter than 1k bases to reduce the file size.

***Note:*** The pre and assembling processes are independently conducted for each paired FASTQ file.

***Optional:*** We advise that the system has sufficient disk space to store all preprocessed FASTQ and assembled FASTA files. The rest of the files, including intermediate and temporary files, can be deleted to save the disk space.

### Extraction of CRISPR direct repeats from the assembled contigs

**Timing: 1–2 h**

From the assembled contigs, discover CRISPR consensus direct repeats.

6. Run CRISPRDetect to discover consensus CRISPR direct repeats.

$ CRISPRDetect.pl -q 0 -f scaffolds.renamed.fasta -o crispr_detect_output -array_quality_score_cutoff 2

7. From the output gff file, extract direct repeats, and convert them into a FASTA file.

$ grep 'repeat_region' crispr_detect_output.gff | cut -f 9 | tr ';' '∖n' | egrep 'ˆNote=' | cut -f 2 -d '=' | awk '{n++; printf(">DR_%i∖n%s∖n", n, $0)}' > crispr_dr.fasta

***Note:*** Again, these direct repeat extractions are independently conducted for each assembled FASTA file.

8. After discovering all direct repeats from each assembled FASTA file, we merge them into a single file, assign unique identifiers, and remove redundancy.

$ cat {all crispr dr fasta files} > merged_crispr_dr.fasta

$ awk '/ˆ>/ {n++; print ">dr_"n; } !/ˆ>/ {print}' merged_crispr_dr.fasta > merged_crispr_dr.renamed.fasta #giving new unique identifiers

$ cd-hit-est -S 1 -c 1 -i merged_crispr_dr.renamed.fasta -o merged_crispr_dr.repr.fasta

***Optional:*** We provided the direct repeat sequences extracted from the human gut metagenomes in the previous study. The sequences are stored in a FASTA formatted file located in the supplementary folder (supplementary_data/crispr/drs/all_dr.clustered.fasta).

### Extraction of CRISPR spacers from the preprocessed reads

**Timing: 1 h**

***Alternatives:*** A pipeline script for the spacer collection is provided in the repository (pipeline_scripts/collect_spacers.sh).

Here, we return to the preprocessed FASTQ files. From them, we extract CRISPR spacers from reads containing CRISPR direct repeats.

9. Extract direct repeat-containing reads. This initial filtering step significantly reduces the number of reads, effectively speeding up the following spacer extraction processes.

$ bbduk.sh in=ecc.fastq.gz ref=merged_crispr_dr.repr.fasta outm=dr_containing_reads.fastq.gz interleaved=t k=21 hdist=1 rename=t

10. Second filter to mask the direct repeats in the reads.

$ bbduk.sh in=dr_containing_reads.fastq.gz ref=merged_crispr_dr.repr.fasta kmask=R k=19 hdist=1 out=dr_masked.fastq mink=15

11. Extract spacers from the masked reads using our python script.

$ {path to the virome_scripts directory}/pipeline_scripts/extract_spacers.py -s {sample name} dr_masked.fastq > spacers.fasta

***Note:*** The python script used above is available from our repository (pipeline_scripts/extract_spacers.py).

***Optional:*** The python program used above does not output short (<20 bp) and long (>50 bp) sequences by default. These parameters can be changed by options (-s and -l).

***Note:*** Spacer extraction is conducted for each preprocessed FASTQ file.

12. Merge all discovered spacers, assign unique identifiers, and remove redundancy.

$ cat {all spacer fasta files} > all_spacers.fasta

$ awk '/ˆ>/ {sub(/ˆ[ˆ[:space:]]+[[:space:]]/, ""); n++; printf(“>spacer_"n"∖t"$0);} !/ˆ>/ {print}' all_spacers.fasta > all_spacers.renamed.fasta #giving new unique identifiers

$ cd-hit-est -T 8 -M 10000 -d 0 -sf 1 -s 0.9 -c 0.98 -i all_spacers.renamed.fasta -o all_spacers.repr.fasta

***Optional:*** It is highly advisable to make each spacer and direct repeat trackable to the original samples, contigs, and/or associated direct repeats. This can be achieved using FASTA descriptions in each record.

***Optional:*** We provided the spacer sequences extracted from the human gut metagenomes in the previous study. The sequences are stored in a FASTA formatted file located in the supplementary folder (supplementary_data/crispr/spacers/all_spacers.clustered.removed_short.fasta).

### Protospacer discovery

**Timing: 1–2 h**

Here, we discover protospacers by aligning the spacer sequences to CRISPR-masked contigs.

13. Search CRISPR direct repeats from the contigs.

$ makeblastdb -dbtype nucl -in scaffolds.renamed.fasta

$ blastn -evalue 1e-5 -task ‘blastn-short’ -outfmt 6 -num_threads 8 -query merged_crispr_dr.repr.fasta -db scaffolds.renamed.fasta > dr.blastn

14. Mask sequences around direct repeat aligned regions.

$ samtools faidx scaffolds.renamed.fasta

$ awk ‘BEGIN{FS="∖t"; OFS="∖t"} $10<$9{t=$9; $9=$10; $10=t} {print $2,$9–1,$10}' dr.blastn | sort -k1,1 -k2,2n | bedtools merge -i /dev/stdin -d 60 | bedtools slop -b 60 -i /dev/stdin -g scaffolds.renamed.fasta.fai | bedtools maskfasta -bed /dev/stdin -fi scaffolds.renamed.fasta -fo crispr_masked.fasta

15. Align spacers to the masked contigs.

$ makeblastdb -dbtype nucl -in crispr_masked.fasta

$ blastn -evalue 1e-5 -perc_identity 93 -task ‘blastn-short’ -outfmt 6 -num_threads 8 -query all_spacers.repr.fasta -db crispr_masked.fasta > spacers.blastn

***Note:*** Protospacer search is conducted for each assembled FASTA file.

16. Merge all discovered protospacers into a single BED-formatted file.

$ cat {all spacer blastn output files} | awk ‘BEGIN{FS="∖t"; OFS="∖t"} $10<$9{t=$9; $9=$10; $10=t} {print $2,$9–1,$10,$1}' | sort -k1,1 -k2,2n > all_protospacers.bed

***Optional:*** We provided the protospacers discovered from the assembled human gut metagenome contigs in the previous study. The protospacers are stored in a BED-formatted file located in the supplementary folder (supplementary_data/targeted_sequences/protospacers/all_protospacers.bed).

### Spacer clustering based on co-occurrence

**Timing: 2–4 h**

Here, we cluster spacers using a co-occurrence network calculated from the spacer alignment result. From this network, we discover the graph communities, representing subsets of spacers that corresponding protospacers co-occur together across the assembled contigs. We extract the protospacer-enriched regions using these graph communities, i.e., spacer clusters. This process effectively reduces the false-positive ratio by excluding lone or randomly scattered protospacers.

17. Initial clustering is based on distance.

$ bedtools cluster -d 50000 -i all_protospacers.bed | awk ‘BEGIN{FS="∖t"; OFS="∖t"} {print $1"_cluster_"$5,$2,$3,$4}' > initial_cluster.bed

18. Calculate a weighted graph from the spacer co-occurrence and generate an abc formatted file.

$ {path to the virome_scripts directory}/graph_clustering/generate_abc_edgefile.py initial_cluster.bed > protospacers.abc

***Note:*** The python script used above is available in our repository (graph_clustering/generate_abc_edgefile.py).

19. Convert the abc file into an mci format.

$ mcxload -abc protospacers.abc --stream-mirror -write-tab data.tab -o protospacers.mci

20. Run an mcl program to discover graph communities.

$ mcl protospacers.mci -I 4 -pi 0.4 -te 8

21. Convert the mcl output to a tabular format.

$ mcxdump -icl out.protospacers.mci.I40pi04 -tabr data.tab > protospacers_cluster.tab

**CRITICAL:** Each tab-delimited line within this output file consists of a spacer cluster.

22. Mark CRISPR-targeted regions using the clustering result and the merged protospacer bed file.

$ {path to the virome_scripts directory}/graph_clustering/mark_crispr_targeted.py all_protospacers.bed protospacers_cluster.tab > crispr_targeted.bed

***Note:*** The python script used above is available in our repository (graph_clustering/mark_crispr_targeted.py).

***Note:*** The fourth column in the output file is formatted as {cluster id}:{protospacer count in the region}.

23. Merge the regions to rescue the fragmented clusters.

$ sort -k1,1 -k2,2n crispr_targeted.bed > crispr_targeted.sorted.bed

$ bedtools merge -d 1000 -i crispr_targeted.sorted.bed -o collapse -c 4 > crispr_targeted.merged.bed

24. Extract CRISPR-targeted regions from the assembled contigs.

$ samtools faidx scaffolds.renamed.fasta

$ cut -f 1,2 scaffolds.renamed.fasta.fai | sort -k 1,1 | join -t $'∖t' - <(sort -k 1,1 crispr_targeted.merged.bed) | awk 'BEGIN{OFS="∖t"; FS="∖t"} $4>$2{$4=$2} {print $1,$3,$4,$5}' | bedtools getfasta -fi scaffolds.renamed.fasta -bed /dev/stdin -fo crispr_targeted.fasta

***Note:*** The extraction of CRISPR-targeted sequences is conducted for each assembled FASTA file.

***Optional:*** We provided the CRISPR-targeted terminally redundant sequences extracted from the assembled human gut metagenome contigs in the previous study. The sequences are stored in a FASTA formatted file located in the supplementary folder (supplementary_data/targeted_sequences/tr/tr_sequences.fasta).

## Expected outcomes

**Figure 1:**
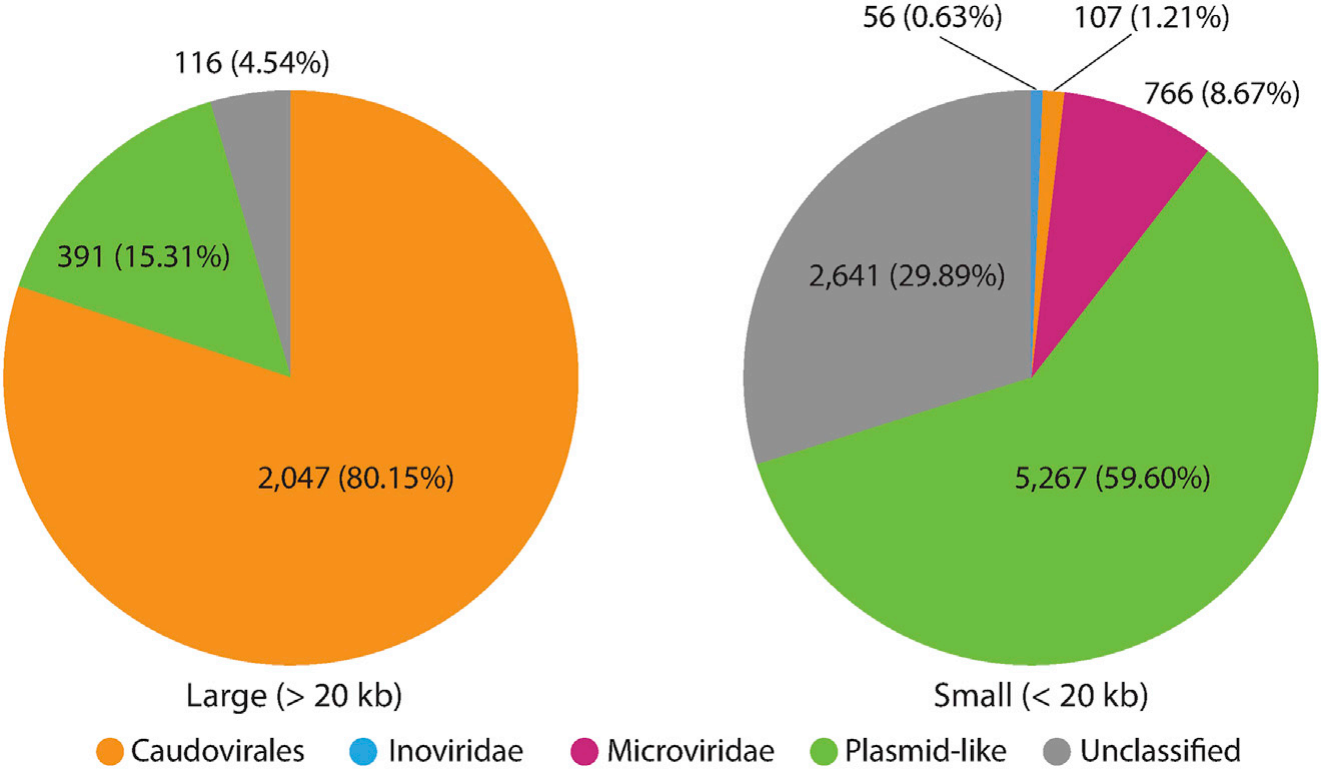
Classification results of CRISPR-targeted terminally redundant sequences from the human gut metagenome The most popular elements among the large (>20 kb) CRISPR-targeted sequences in the human gut metagenome were tailed phages. Conversely, ssDNA viruses, plasmid-like elements, and many unclassified sequences were common among the small (<20 kb) sequences. This result was obtained from our previous study (Sugimoto et al., 2021).

The final output FASTA files contained CRISPR-targeted sequences. These sequences typically range in size between a few hundred and several hundred thousand bases. The output sequences likely contain multiple kinds of MGEs (Figure 1).

## Quantification and statistical analysis

• Sequence clustering should be conducted to remove redundancy and fragmentations from the final output sequences.
• Genomic completeness could be investigated by checking terminal redundancy or inverted terminal repeats. In the repository, we provided python scripts that extract terminally redundant or inverted repeat sequences from a FASTA file (utils/circular_contigs.py and utils/inverted_repeat_contigs.py).
• CRISPR-Cas systems target various elements, including viruses, plasmids, transposons, and chromosomes. A protein homology search is a common method to classify the novel genomes. We suggest using a high-sensitivity homology search method based on hidden Markov models.

## Limitations

It is possible to predict the CRISPR-targeting hosts by searching protospacer-associated direct repeats in the reference genome database. However, CRISPR undergoes intense horizontal gene transfer. Therefore, combining it with other methods, such as tRNA alignment, is highly advisable to predict the infecting host.

## Troubleshooting

### Problem 1

Genome assembly takes time (step 4).

### Potential solution

The time metagenome assembly takes strongly varies between libraries. If an assembly takes longer than a day, and the library size is very large (>100 Gb), one can downsample the FASTQ file by randomly selecting pairs from the original files or normalizing the depth *in silico*. Both approaches can reduce the file size and potentially the assembling time.

### Problem 2

Why did you skip the error-correction step in the SPAdes program (step 4)?

### Potential solution

We found that the SPAdes program is sometimes trapped in the error-correction step and seems to endlessly run for more than four or five days. This phenomenon is sporadic, hard to replicate, and might depend on the system specifications. Such a problem was highly problematic when we were analyzing more than a thousand samples. To avoid this issue, we skipped the error-correction step in the SPAdes program and instead used the tadpole program for the preassemble error correction. We compared the statistics between the assembled contigs, using SPAdes or tadpole, and found no significant difference between them.

### Problem 3

Assembled contigs are short (step 4).

### Potential solution

The less populated organisms in the sample are likely to be shallowly sequenced, leading to highly fragmented and partial contigs. Therefore, they might require deeper sequencing to assemble the complete genome. Conversely, the metadata of SRA recorded libraries could be wrong sometimes. For example, we encountered several libraries likely produced from 16S amplicon sequencing but recorded as a whole genomic sequencing. It is advisable to avoid such libraries and/or refer to the experimental method described in the original article if necessary.

### Problem 4

The constructed spacer co-occurrence network is small and fragmented (step 21).

### Potential solution

In the original study, we used millions of spacers extracted from thousands of human gut metagenome datasets to construct a network. A significant number of spacers and spacer-aligned contigs are required to build a versatile network and predict graph communities. If you are attempting to analyze other than the human gut metagenome using a few materials, it might be advisable to skip the network building process entirely and manually check each protospacer containing contigs.

### Problem 5

Protein homology searches do not hit the database (quantification and statistical analysis).

### Potential solution

Many MGE encoded protein sequences such as capsids and polymerases are extremely diversified, therefore it is often difficult to detect their homology to known sequences using the pairwise alignment-based method which is adopted by such as the BLAST program. In order to overcome this issue, hidden Markov models (HMMs) based programs such as HMMER (Eddy, 2011) or HH-suite (Steinegger et al., 2019) could be used for the higher sensitivity homology search. Using the predicted protein sequences from the discovered CRISPR-targeted sequences as seeds, one could iteratively search the bulk of metagenome-derived protein sequence databases such as Metaclust (Steinegger and Söding, 2018). The aligned sequences were used to build an HMM, which is used as a query to search a curated protein database such as Protein Data Bank.

## Resource availability

### Lead contact

Ituro Inoue: itinoue@nig.ac.jp.

### Materials availability

CRISPR spacers and direct repeats extracted in the previous study are available from our repository.

## Data Availability

The source codes used in this protocol are available from our repository.
